# Analysis of renal artery morphometery in adults: A study conducted by using Multidetector computed Tomography Angiography

**DOI:** 10.12669/pjms.334.13063

**Published:** 2017

**Authors:** Maria Mohiuddin, Arsalan Manzoor, Muhammad Ali, Nuzhat Hassan

**Affiliations:** 1 Dr. Maria Mohiuddin, Senior Lecturer, Department of Anatomy, Ziauddin University, Hospital, Karachi, Pakistan; 2 Dr. ArsalanManzoor, Assistant Professor, Department of Anatomy, Ziauddin University, Hospital, Karachi, Pakistan; 3 Dr. Muhammad Ali, Assistant Professor, Head of Department of Radiology, Ziauddin University, Hospital, Karachi, Pakistan; 4 Prof. Dr. Nuzhat Hassan, Chairperson, Department Of Anatomy, Ziauddin University, Hospital, Karachi, Pakistan

**Keywords:** Age, Computerized Tomography, Gender, Renal artery

## Abstract

**Objective:**

To determine a reference range of renal artery measurements by using Multidetector Computed Tomography (MDCT) angiography and to find association of renal artery measurements with side of artery, gender and age.

**Method:**

Two hundred and fifty study participants without renal artery disease who werepresented to Radiology Department, Ziauddin Hospital, Karachi, from November, 2016 to April, 2017 were included in this study. Main renal artery measurements were taken on Multidetector computed angiography and variation with side, gender and age were analyzed. Statistical analysis was done on Statistical Package for Social Sciences (SPSS) version 20. Independent sample T test, one way ANOVA and Pearson’s correlation analysis were applied. P-value of < 0.05 was considered significant.

**Results:**

A significance difference (p=0.001) was seen between mean right renal artery (diameter 6.66 ± 0.39 mm; length 44.69 ± 2.48 mm) and left renal artery (diameter 6.79 ± 0.36; length 35.10 ± 2.86 mm). Females found to have smaller mean diameter and length of renal arteries than males. However, a weak negative correlation was seen between mean renal artery diameter and age (right r= -0.158, p=0.0121; left r= -0.017, p= 0.708).

**Conclusion:**

The mean diameter and mean length were found to be significantly different between right and left main renal artery and between males and females. A significant weak negative correlation was observed between renal artery diameter and age.

## INTRODUCTION

In South Asian country like Pakistan, incidence of end stage renal disease (ESRD) is gradually increasing and has produced a large economic burden.^1^ For end stage renal disease, renal transplant is considered as the best choice of treatment modality. It is essential, that each potential living donor should have a complete renal vasculature evaluation to avoid complications.^2^ It is important for the surgeon to know the precise range of diameter, length and exact site of origin of renal arteries for accurate renal assessment.^3^ Due to increase in radiological imaging techniques, normal range of renal artery diameter is very important for guiding interventional radiologist during procedures like arterial catheterization andangioplasty.^4^ Decrease in luminal diameter of renal artery leads to renal artery stenosis that will causerenovascular hypertension and ischemic nephropathy.^5^ Percutaneous arterial stenting is the choice of treatment for renal artery stenosis, but this requires definitemeasurement of renal artery diameter and length.^6^-^8^

Renal arteries arisesnormally from lateral aspects of abdominal aorta at level of intervertebral disc between 1^st^ and 2^nd^ lumbarvertebrae’s below the origin of superior mesenteric artery.^9^ Arteries run laterally and enter the renal hilum and divides into segmental branches.^10^ Variation in renal artery dimensions was frequently seen in routine clinical and surgical practice. As renal artery measurements varies with factors like age, gender and geographical location. Studiesreportedvariation inrenal artery dimensions gender, age and side of artery.^11^,^12^

Conventional catheter angiography is regarded as a gold standard technique to visualize arteriesbutits invasive nature limits its use. In contrast, Multidetector computed tomography (MDCT) angiography is considered to be a major advancement in computerized tomography (CT) imaging.^13^,^14^ It has many advantages as high speed acquisition, accurate, less invasive, low cost as compared to magnetic resonance angiography (MRA)andprovides multiple three dimensional reconstructed images that will help to see accurate renal vascular anatomy in detail and alsovisualization of surrounding renal parenchyma.^15^

Studies have been performed in different parts of worldto establish normal reference range of renal artery dimensions.^3^,^16^,^17^ Review of literature showed that studies performed in our population were mainly in context with renal vascular pathologies as renal artery stenosis and renovascular hypertension.^18^-^20^ Thus, this study was conducted to determine a reference range of normal renal artery measurements and to find itsvariation with side of artery, gender and age in our population by using MDCT angiography in our population.

## METHODS

Study subjects were recruited for those individuals who were presented to Radiology Department of Ziauddin University Hospital, Clifton campus, Karachi, from November 2016 to April 2017 for abdominal contrast CT examination without renal diseases. Study was conducted after approval from Ethical review committee of Ziauddin University. A total of 250 individuals aged 21 years to 60 yearswere recruited through consecutive sampling. Informed consent and clinical history was obtained from all participants. Those with serum creatinine ≤ 1.3 mg/dl (as per Hospital Lab value) and adults with no known renal artery and vein diseases were included. Participants with a history of renal transplant, renal surgery, vasculitis, congenital vascular anomalies, allergic reaction to contrast agent, pregnancy, hypertension and diabetes mellitus were excluded.

CT scan was performedusing standard protocol,^14^,^16^ ona 16-slice MDCT scanner (Toshiba 16 slicer Alexion, Japan). Prior to scan emptiness of stomach and bowels were ensured.^21^ Contrast material was given through a wide bore intravenous line placed in the antecubital fossa at the rate of 4 ml/sec and amount of 2 ml/kg contrast was injected. The patient was instructed to hold his/her breath for 10 seconds and scan was initiated. The scanned area was extended from diaphragm to iliac crest. Acquisition of image data was initiatedafter a preset delay of 10 to 15 secondsafter the start of the contrast agent injection. Image data was transferred to animaging workstation (Toshiba Medical Systems), which was used to post-process volumetric MDCT data. Special computer software was used for3-dimensional reconstruction of images in different planes and projections in arterial phase of scan. Multiplanar reconstructed (MPR) and Maximum intensity projection (MIP) images with thin (0.5 mm) and thick (3 mm) slice thicknesses were used to evaluate the renal arteries. Oblique coronal and axial MIP images with curved planar reconstruction were generated to visualize renal artery along its route. Renal artery diameter and length were measured. Diameter was measured in the proximal segment (1.5 cm from origin) of the renal artery.^22^

Data was analyzed on SPSS version 20. Quantitative variables were presented as mean ±standard deviation. Quantitative variables were compared by using one sample t-test, independent t-test and one way ANOVA. Correlation analysis by using Pearson’s correlation was applied to test the relationship between variables. P-value < 0.05 was considered significant.

## RESULTS

Out of 250 individuals 129 (51.6%) were males and 121(48.4%) were females. Study participants were 21 to 60 years of age. Mean age of males were 43.5 ± 11.0 years and females were 44.3 ± 12.69 years. Mean values of right and left renal arteries are shown in Table-I. A significant difference (p= 0.001) of mean diameter and length was observed between right and left main renal arteries (Table-I).

**Table-I:** Comparison of Right and Left Renal Artery.

*Parameter*	*No. of Participant (n)*	*Right Renal Artery Mean ± SD*	*Left Renal Artery Mean ± SD*	*p-value*
Diameter (mm)	250	6.66 ± 0.39	6.79 ± 0.36	0.001*
Length (mm)	250	44.69 ± 2.48	35.10 ± 2.86	0.001*

Confidence interval 95%,

* p-value ≤ 0.05 is significant

**Fig. 1:**
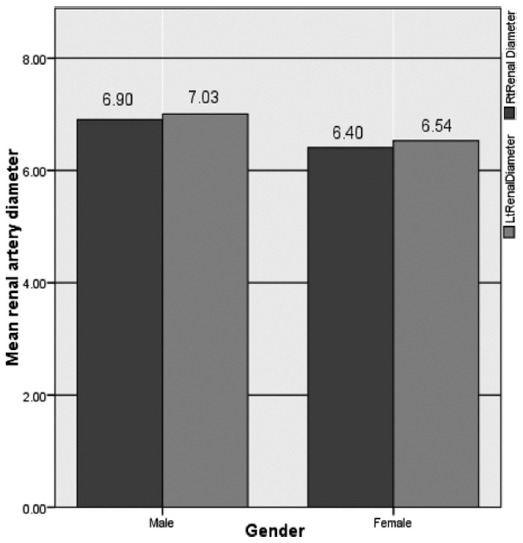
Comparison of mean diameter of right renal artery and left renal artery in males and females

In males mean diameter of right renal artery was 6.90±0.24 mm and left renal artery was 7.03±0.22mm. In females mean diameter of right renal artery was 6.40 ± 0.35 mm and of left renal artery was 6.54 ± 0.31 mm. A significant difference (p = 0.001) of mean diameter of right and left renal arteries was observed between males and females (Fig. 1). Moreover in males mean right renal artery length was46.45 ± 1.93mm and left renal artery length 36.54 ±1.83mm were significantly different (p=0.001) from mean right renal artery length 42.81 ± 1.39mm and left renal artery length 33.56 ± 2.96 mm in females.

All participants were stratified accordingly into four age groupsi.e. Group-1 (21 to 30 years), Group-2 (31 to 40 years), Group-3 (41 to 50 years) and Group-4 (51 to 60 years). Mean diameter of right renal and left renal arteries were found significantly different (p=0.001) among age groups. By using Pearson’s correlation analysisa significant weak negative correlation (r= - 0.158, p= 0.012)was found between mean right renal artery diameter and age. Aweak negative correlation (r= -0.017, p=0.708) was foundbetween mean left renal diameter and age. Moreover no significant difference of mean length of right and left renal arteries was observed between age groups (Table-II).

**Table-II:** Comparison of mean renal artery diameter and length in different age groups.

*Age groups (years)*	*1 (21-30)*	*2 (31-40)*	*3 (41-50)*	*4 (51-60)*	*P-value*
Total (n)	n=45	n=46	n=78	n=81
Rt Renal Dia(mm)	6.63 ± 0.18	6.94 ± 0.19	6.95± 0.21	6.26 ± 0.30	0.001*
Lt Renal Dia(mm)	6.75 ± 0.16	7.05 ± 0.17	7.07 ± 0.22	6.43 ± 0.27	0.001*
Rt Renal L(mm)	44.68 ± 2.29	44.87 ± 2.92	45.26 ± 2.11	44.07 ± 2.59	0.08
Lt Renal L(mm)	35.24 ± 2.45	34.85 ± 4.88	35.49 ± 2.24	34.78 ± 2.25	0.58

Rt: right, Lt: left, Dia: diameter, L: length, Confidence interval 95%,

* p-value ≤ 0.05 is significant

## DISCUSSION

It is essential for the radiologists and surgeons to have background knowledge of normal range of renal artery measurements and variation in measurementsin a specific population as this can affect complex and expertise required procedure.^23^,^24^ In our study a significant difference was observed between mean diameter of right and left main renal arteries. Mean diameter of right renal artery was found to be smaller than mean diameter of left renal artery. Our results are in agreement with the results reported in a study conducted in Columbia, whichreported right renal artery diameter to be significantly less than left renal artery diameter.^25^ A study conducted in Iran by using Multi-slice CT scan reported right renal artery diameter to be smaller than left renal artery diameter (p=0.35).^26^ Mean length of right renal artery in present study was found to be significantly (p=0.001) longer than mean length of left renal artery (Table-I). A study conducted on 200 renal pedicles in Brazilreported mean right renal artery length longer thanleft renal artery length (p=0.002).^16^ This is probably due to the location of abdominal aorta to the left of median plane in abdomen and the longer path of the renal artery on the right side, as suggested by different authors.^3^,^16^,^25^

In this study, females were found to have significantly (p= 0.001) smaller mean diameter and mean length of right and left renal arteries as compared to males (Table-I). A novel study conducted in university of Virginia reportedbilateralrenal arteries more in diameter and length in males as compared to females (p<0.001) and concluded, these variation in measurements arebecause of relatively large body size of males as compared to females.^12^

Aging is associated withdecreased vascular compliance and increased vascular rigidity.^27^ In this study, a 0.3 mm increase in mean diameter of both renal arteries was seen after 20 years of age and an approximately constant diameter during fourth and fifth decades of life. However, a decrease of 0.64 mm was seen after 50 years of age. Aweak negative correlation was seen between mean right renal artery diameter (r = - 0.158, p= 0.012) and left renal artery diameter (r = - 0.017, p = 0.708)with age. Moreover, no significant difference was observed between length ofrenal arteries and age. Our findings are in accordance with results reported in a study conducted in South Africa, in whicha 0.4mm increase in luminal diameter was observed after second decadethat remain almost constant till fifth decade and a subsequent decrease of 0.4 mm after 60 years of age.^11^ Another study also reported a strong influence of increasing age on narrowing of renal artery diameter.^28^ Increase in diameter during adulthood is probably due to increased physical activity and associated increased cardiac output.^29^ While, a decrease in luminal diameter of artery with advancing age is possibly due to progressive thickening of the tunica intima layer, separation of individual elastin lamellae and increase in collagen matrix in arterial wall.^27^

In this study we have established a normal reference range regarding renal artery dimensions in adults in our local population. Morphometeric data regarding renal arteries and variation in measurements is imperative for selecting donors for renal transplant, to diagnose renal artery stenosis, guiding the radiologist during arterial catheterizations, laparoscopic nephrectomies, to design and place arterial stent grafts.^3^,^28^,^30^,^31^

## CONCLUSION

Our study concluded that significant difference of diameter and length exists between right and left renal arteries. Renal artery diameter and length are significantlydifferent between males and females. A significant weak negative correlation was observed between renal artery diameter and age. However, large scale multicentre nationwide studiesshould be done in future to further strengthen our result.

### Author’s Contribution

**MM** designed, did data collection and writes the manuscript.

**AM** did critical analysis and editing of manuscript.

**MA** provided intellectual and clinical approval.

**NH** did critical analysis and provides intellectual support.
